# Development and implementation of a core genome multilocus sequence typing scheme for *Yersinia enterocolitica:* a tool for surveillance and outbreak detection

**DOI:** 10.1128/jcm.00040-24

**Published:** 2024-07-11

**Authors:** Joao Pires, Lin T. Brandal, Umaer Naseer

**Affiliations:** 1 Department of Infection Control and Preparedness, Norwegian Institute of Public Health, Oslo, Norway; 2 ECDC Fellowship Programme, Public Health Microbiology path (EUPHEM), European Centre for Disease Prevention and Control (ECDC), Stockholm, Sweden; Universitat Munster, Münster, Germany

**Keywords:** cgMLST, molecular typing, outbreak, *Yersinia enterocolitica*, yersiniosis

## Abstract

*Yersinia enterocolitica* (*Y. enterocolitica*) is the most frequent etiological agent of yersiniosis and has been responsible for several national outbreaks in Norway and elsewhere. A standardized high-resolution method, such as core genome Multilocus Sequence Typing (cgMLST), is needed for pathogen traceability at the national and international levels. In this study, we developed and implemented a cgMLST scheme for *Y. enterocolitica*. We designed a cgMLST scheme in SeqSphere + using high-quality genomes from different *Y. enterocolitica* biotype sublineages. The scheme was validated if more than 95% of targets were found across all tested *Y. enterocolitica*: 563 Norwegian genomes collected between 2012 and 2022 and 327 genomes from public data sets. We applied the scheme to known outbreaks to establish a threshold for identifying major complex types (CTs) based on the number of allelic differences. The final cgMLST scheme included 2,582 genes with a median of 97.9% (interquartile range 97.6%–98.8%) targets found across all tested genomes. Analysis of outbreaks identified all outbreak strains using single linkage clustering at four allelic differences. This threshold identified 311 unique CTs in Norway, of which CT18, CT12, and CT5 were identified as the most frequently associated with outbreaks. The cgMLST scheme showed a very good performance in typing *Y. enterocolitica* using diverse data sources and was able to identify outbreak clusters. We recommend the implementation of this scheme nationally and internationally to facilitate *Y. enterocolitica* surveillance and improve outbreak response in national and cross-border outbreaks.

## INTRODUCTION

Yersiniosis is the third most frequent food-borne zoonosis reported in Europe (1) and fifth in the United States (2). While this disease is generally self-limiting, and antimicrobial therapy is not usually necessary, 33% of yersiniosis cases reported in 2021 from the European Union/European Economic Area (EU/EAA) required hospitalization (1). *Yersinia enterocolitica* (*Y. enterocolitica*) is the most frequent etiological agent of yersiniosis, accounting for 98.1% of all reported cases in the EU/EAA. Pigs are the main reservoir of human pathogenic *Y. enterocolitica* (3). Therefore, the consumption of pork, pork-containing food-stuffs, and cross-contaminated products constitute risk factors for yersiniosis (3, 4) and have been the source of several outbreaks in multiple countries (5). Nonetheless, outbreaks of *Y. enterocolitica* have increasingly been associated with vegetable greens (6, 7).

Traditionally, typing of *Y. enterocolitica* relied on biochemical reaction biotyping and O-antigen serotyping, which classified the species into six biotypes (8, 9)—the non-pathogenic 1A, and the pathogenic 1B, 2, 3, 5, and 6, and over 50 serotypes (10). To date, 11 serotypes have been described as pathogenic to humans (11). Globally, O:3 is the most frequent human pathogenic serotype (12, 13). In Europe, the predominant serotypes include O:3, O:5–27, and O:9, whereas in the United States, it is mainly O:8 (3). Further discriminating strains within this species has been achieved using pulsed-field gel electrophoresis, multiple locus variable number of tandem repeats analysis, and multilocus sequence typing (MLST) (14). Although these methods have proven useful in identifying outbreaks and allowed for the identification of sublineages within serotypes (15, 16), their limitations include low interlaboratory comparability and/or low resolution (14). Hence, a transition to next-generation sequencing (NGS)-based methods, such as core-genome MLST (cgMLST), will provide a standardized typing system that will enable subtyping at a higher granularity. This will improve data comparability across geo-temporal scales, which is fundamental for detection and investigation of both national and cross-border outbreaks, as well as improving surveillance of this pathogen.

To date, two cgMLST schemes have been developed for *Yersinia* spp. and have been useful in identifying lineages within different species, including *Y. enterocolitica* (8, 17). However, as both schemes use gene targets available across multiple species, the specificity for *Y. enterocolitica* is potentially reduced, which can lead to poor performance in discriminating strains and identifying outbreaks where increased discriminatory power is needed (18). Furthermore, given the potential health and economic impact of *Y. enterocolitica*, developing a specific cgMLST scheme with high discriminatory power will be important for effective surveillance and accurate outbreak detection and investigation of this pathogen.

In this project, we aim to develop a cgMLST scheme for *Y. enterocolitica* that can form the basis of a standardized nomenclature for whole-genome sequence-based *Y. enterocolitica* typing. First, we defined a gene set of *Y. enterocolitica* core genome representing the genetic diversity within the *Y. enterocolitica* population based on well-characterized strains, and second, we challenged this scheme’s ability to discriminate using alternative schemes and genomes by different assembly methods. Finally, we assessed its suitability for outbreak detection using isolates from known outbreaks and sporadic cases.

## MATERIALS AND METHODS

### Isolates

Yersiniosis is a mandatory notifiable disease in Norway, and isolates of all laboratory-confirmed cases of yersiniosis caused by *Y. enterocolitica* or *Y. pseudotuberculosis* are sent to the National Reference Laboratory for Enteropathogenic Bacteria (NRL) at the Norwegian Institute of Public Health (NIPH). Also, other *Yersinia* spp. are sent to the NRL if *Y. enterocolitica* or *Y. pseudotuberculosis* cannot be excluded. Since mid-2018, all *Yersinia* spp. isolates have been sequenced using Illumina NGS technology at the NRL. For the purposes of this study, we have used a total of 920 *Yersinia* spp. genomes. These encompass all available *Yersinia* spp. genomes at the NRL*—Y. enterocolitica* (*n* = 561), *Y. frederiksenii* (*n* = 9), *Y. bercovieri* (*n* = 7), *Y. aleksiciae* (*n* = 4), *Y. intermedia* (*n* = 3), *Y. mollaretii* (*n* = 3), *Y. kristensenii* (*n* = 2), *Y. rohdei* (*n* = 2), *Y. aldovae* (*n* = 1), and *Y. massiliensis* (*n* = 1)—as well as 327 *Y. enterocolitica* available through the National Center for Biotechnology Information (NCBI) Assembly Database by 21 November 2022 (19). A description of these isolates can be found in Table S1.

### Whole-genome sequencing

Whole-genome sequencing of all received *Y. enterocolitica* isolates at NRL was performed according to the following protocol: DNA extraction was performed by MagNAPure 96 (Roche Molecular Systems Inc., Pleasanton, US). KAPA HyperPlus (Kapa Biosystems, Wilmington, US) was used for library preparation and Agencourt AMPure XP (Beckmann Coulter Life Sciences, Indianapolis, US) for removal of adaptor dimers. WGS was performed as paired-end sequencing on the NextSeq or MiSeq (Illumina, Inc., San Diego, US) platform aiming for a coverage of >50× . Quality control of the raw reads was done through FastQC. All sequences have been submitted to the European Nucleotide Archive and are available through BioProject PRJEB67986.

### Development of a cgMLST scheme

We developed the cgMLST scheme using the cgMLST Target Definer within the Ridom SeqSphere + software version 8.5.1 (20). This tool performs a genome-wide gene-by-gene comparison to identify all gene targets within a reference genome. Thereafter, it uses a set of reference genomes (also called seed genomes) to identify which genes to include in the scheme. These will be genes found once in each of the reference genomes with at least 90% sequence identity and 100% overlap, and have correct start and stop codons as per default settings of the cgMLST Target Definer Tool (21). All genes not meeting these criteria are removed from the scheme.

For the development of this scheme, we used NC_008800.1 (serotype O:8, biotype B1) as our reference genome and 15 high-quality query genomes (22). Query genomes were selected to represent the genetic diversity *of Y. enterocolitica*. To pick genetically diverse isolates, we downloaded all genomes available marked as “Complete” and “Chromosome” in the *Y. enterocolitica* NCBI genome database and calculated the whole-genome mash distance using PATO R package (23). Mash distances were then used to generate a phylogenetic tree. The 15 available genomes were chosen to be representative of the different branches of this tree. We used this approach since the metadata available on NCBI did not provide information on the serotype for all genomes or biotype sublineage as defined by Savin et al. (8).

The list of query genomes can be found in Table S2. The parameters to include a gene from the reference included:

1. A minimum length filter that discards all genes shorter than 50 bp;
  a. A start codon filter that discards all genes that contain no start codon at the beginning of the gene;
  b. A stop codon filter that discards all genes that contain no stop codon or more than one stop codon or that do not have the stop codon at the end of the gene;
  c. A homologous gene filter that discards all genes with fragments that occur in multiple copies within a genome (with identity of 90% and >100 bp overlap);
2. A gene overlap filter that discards the shorter gene from the cgMLST scheme if the two genes affected overlap >4 bp.

The remaining genes are used in a pairwise comparison with BLAST version 2.2.12 to extract the final target genes, within the SeqSphere + software:

1. For processing options, “Ignore contigs shorter than 200 bases”;
2. For scanning options, “Matching scanning thresholds for creating targets from assembled genomes” with “required identity to reference sequence of 90%” and “required alignment to reference sequence with 100%”;
3. For BLAST options, word size 11, mismatch penalty −1, match reward 1, gap open costs 5, and gap extension costs 2. In addition, the target genes will be assessed for quality, i.e., the absence of frame shifts and ambiguous nucleotides.

To check the diversity included in our seed genomes, we applied the scheme on all isolates and obtained an allelic distance matrix in SeqSphere. We used this matrix to create an unweighted pair group method with arithmetic mean tree and annotated biotype sublineages using ggtree (24). We putatively assigned all isolates falling within the same branch of the tree to the respective biotype (Fig. S1).

### Validation of the cgMLST scheme

**Fig 1:**
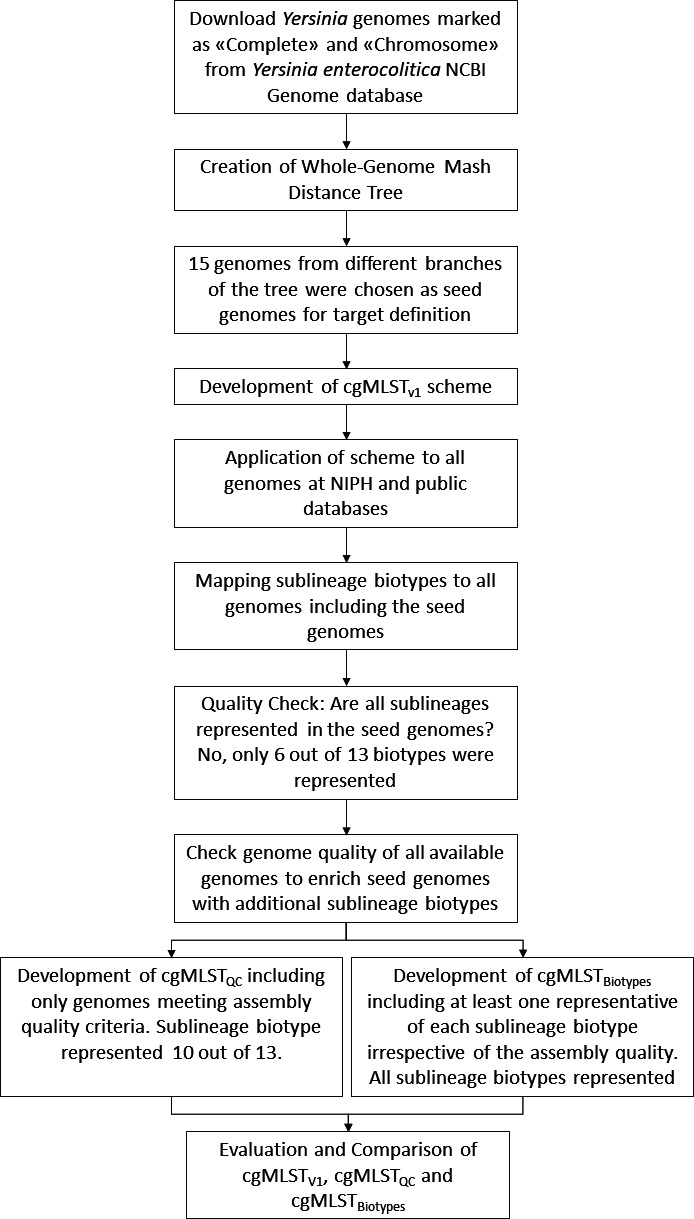
Flowchart of the process of development of a stable cgMLST scheme for *Y. enterocolitica*.

After annotating the biotype sublineages, we identified that the seed genomes used in the development of the cgMLST scheme (hereafter cgMLST_V1_) included six of the 13 biotype sublineages of *Y. enterocolitica* as defined by Savin et al. (8). Therefore, to validate and assure the longevity of this scheme, we developed two additional schemes following the same criteria for target definer but with additional seed genomes (Fig. 1). (i) The first scheme balanced the genetic diversity with genome quality (hereafter cgMLST_qc_) and included at least one seed genome from each biotype if it passed the following assembly quality criteria defined by the World Health Organization (WHO) and European Food Safety Authority (EFSA): assembly with less than 250 contigs, at least 30 times coverage, and N50 of at least 30,0000 (25, 26). This scheme used 26 seed genomes and included 10 of the 13 biotype sublineages. (ii) The second scheme was built on the cgMLST_qc_ and added three genomes (*n* = 29), one per remaining biotype not currently represented in the previous scheme irrespective of their assembly quality (hereafter cgMLST_Biotypes_). The characteristics of used genomes (ST and biotype) for all cgMLST schemes are presented in Table S2.

### cgMLST scheme evaluation

We applied the three cgMLST schemes on all *Yersinia* spp. isolates available at the NRL and those retrieved from NCBI. The cgMLST schemes were assessed according to the following performance criteria: (i) at least 99% of the tested *Y. enterocolitica* needs to have at least 90% of the cgMLST gene targets, (ii) at least 95% of the cgMLST genes are present in 95% of the tested isolates, (iii) these conditions are not met by other *Yersinia* spp., and (iv) the final cgMLST should perform well across most biotypes (the number of genomes available for many biotypes was below 10, and a cut-off at the biotype level was therefore difficult to assign).

Points (i) and (ii) will provide a measure of the presence of the chosen core targets in the population. We compared the percentage of good targets across the three developed schemes using Wilcoxon rank sum and a *post-hoc* Dunn test accounting for multiple comparisons.

To evaluate the stability of the schemes, we analyzed genomes subjected to the following three different assembly methods: SPAdes (27), SKESA (28), and Velvet (29). All data were extracted from SeqSphere + and imported to R version 4.2.1 (30), and analyzed with the *tidyverse* and *rstatix* packages (31, 32).

### cgMLST performance in outbreak detection and surveillance

We evaluated the performance of the developed cgMLST scheme against isolates included as part of outbreak investigations in Norway. We included available sequences from nine different outbreaks, including the following: (i) O:9-ST12 national outbreak in 2011 involving 21 individuals (*n* = 5 sequences available) where suspected vehicle was pre-packed salad mix; (ii) O:9-ST12 2014 outbreak in a military involving 133 individuals, of which 117 who worked at different military camps, where suspected vehicle was pre-packed salad (*n* = 3 sequences available); (iii) O:9-ST12 2018 national outbreak, where suspected vehicle was pre-packed leafy salad (*n* = 20); (iv) O3-ST18 outbreak in December 2019 to January 2020 for which no source was identified (*n* = 11); (v) O:3-ST18 2020 outbreak for which the source was not confirmed, but spinach was the suspected vehicle (*n* = 25); (vi) O:3-ST18 outbreak in December 2020 associated with ready-to-eat-salads (*n* = 10); (vii) O:3-ST12 national outbreak in 2021 (*n* = 17) for which no source was identified; (viii) O:3-ST18 outbreak involving 37 individuals of which 33 were linked to a boarding school in February of 2022 associated with pork consumption (*n* = 13 available sequences); and (ix) O:3-ST18 national outbreak in June/July 2022 potentially associated with salad consumption (*n* = 9). We extracted all genomes from the different outbreaks and identified the maximum number of pairwise allelic differences (AD) within each outbreak. This was used to evaluate how many different thresholds of single-linkage clustering (SLC) algorithm should be performed. SLC is a hierarchical clustering method, which is commonly used within public health institutes and by many databases hosting cgMLST to define and maintain stable complex types (CT). The number of AD required in the SLC to identify an outbreak was defined as the strictest level at which all outbreak isolates were grouped into the same CT. SLC was also used to create a stable numbering system for CTs at different AD thresholds.

The identified threshold was then used to screen the NIPH database for potentially missed outbreaks. According to the current practice at the NIPH for other Enterobacterales, an alert of a potential outbreak is generated when three isolates from non-travel-related cases with the same CT are identified within a period of 30 days. Thus, we created a rolling window of 30 days and identified all CTs at this outbreak threshold that had at least three isolates. The epidemiological characteristics of these isolates were then manually inspected. Finally, we used the CT threshold to identify major CTs within genomes from NRL andNCBI.

### Comparison with published schemes

We compared the final developed scheme with the two published cgMLST schemes that have been developed for *Yersinia* spp. (8, 17). We downloaded the available schemes from Enterobase and Pasteur and imported them into SeqSphere and ran all 619 *Y. enterocolitica* isolates with the same parameters as the final developed scheme (cgMLST_V1_). Additionally, we compared the three schemes using the same criteria as described in the scheme evaluation and performance in outbreak detection. Finally, we compared the SLC cluster assignment at different thresholds. First, we computed the number of unique SLC clusters obtained at different thresholds. Second, we mapped the cluster assignment by identifying at which SLC there is the maximum adjusted Rand index between two schemes using the package *aricode* (33). The adjusted Rand index measures the agreement between cluster assignment by two methods, for which 1 indicates perfect agreement and 0 no agreement. The SLC up to 13 allelic differences were used for this comparison to be able to include clustering between five and 10 differences, which can be used for case finding and case confirmation in multi-country outbreaks (34–36).

## RESULTS

### cgMLST validation

Three different cgMLST schemes were developed based on genome quality and representativeness criteria. The first scheme was designed based on genetically diverse genomes marked as complete or chromosome in NCBI (cgMLST_V1_) and consisted of 2,582 gene targets representing 59.9% of gene content of the reference genome. The second scheme, included genomes from different biotypes passing certain assembly quality criteria (cgMLST_QC_), yielded 2,334 targets (54.1% of the reference genome), and the final scheme contained at least one representative of each biotype (cgMLST_Biotypes_), resulting in 2,277 targets (52.8% of the reference genome).

**Fig 2:**
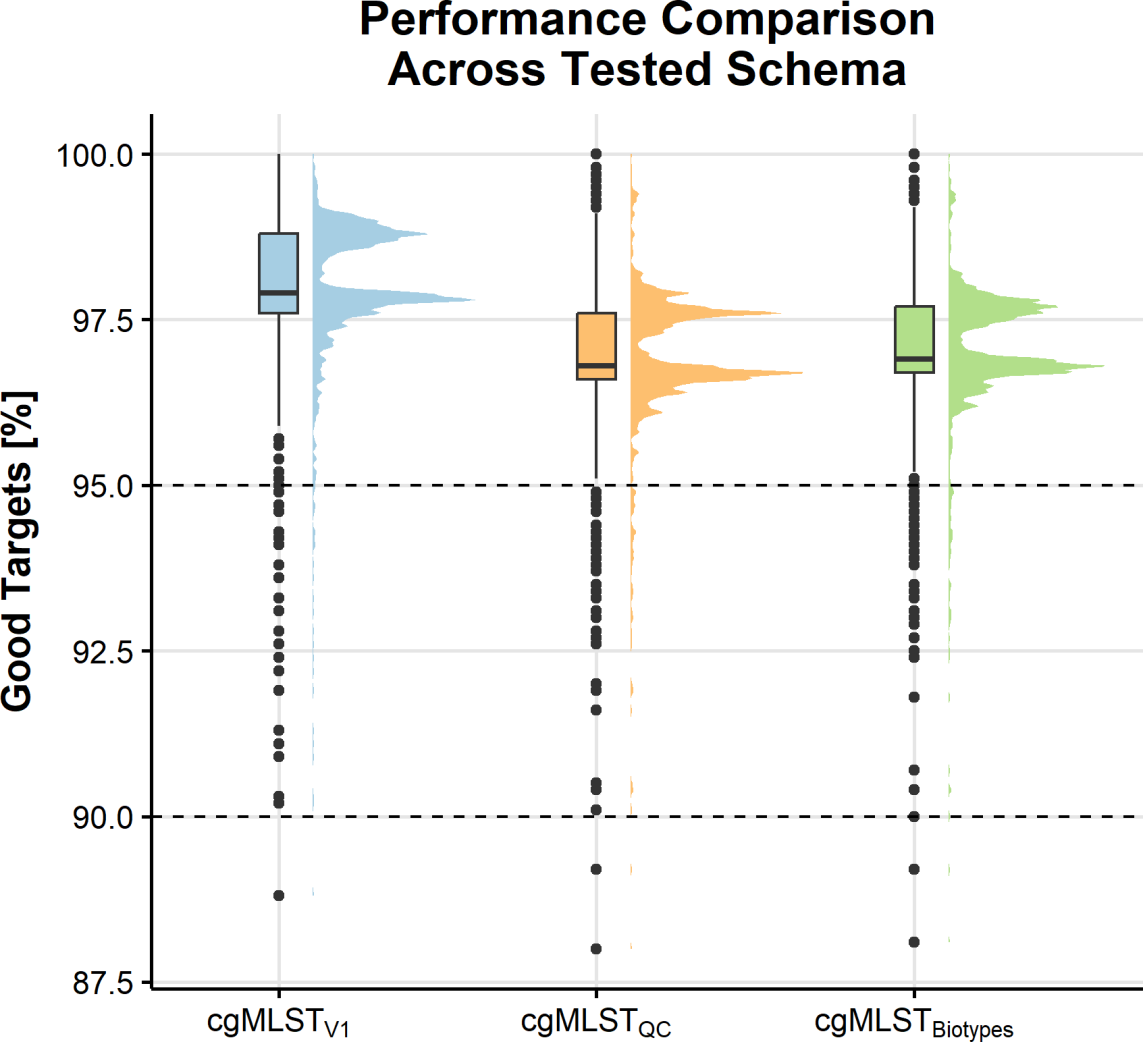
Performance comparison of percentage good targets identified across the three developed cgMLST schemes (cgMLST_V1_, cgMLST_QC_, and cgMLST_Biotypes_). Three outliers fall below the 85% mark in all schemes. The cgMLST_V1_ scheme has 2,582 targets and a median of 97.9% (IQR 97.6%–98.8%) good targets; the cgMLST_Biotypes_ scheme has 2,277 targets and a median 96.9% (IQR 96.7%–97.7%) good targets; the cgMLST_QC_ scheme included 2,234 gene targets and a median 96.8% (IQR 96.6%–97.6%) good targets.

Across all schemes, more than 99% of the tested genomes had at least 90% of the gene targets: 99.9% for cgMLST_V1_ and 99.8% for both cgMLST_Biotypes_ and cgMLST_QC_. However, only cgMLST_V1_ and cgMLST_Biotypes_ achieved more than 95% of the tested isolates with more than 95% of the targets (97.3% and 95.3%, respectively), whereas cgMLST_QC_ achieved this for 94.9% of tested isolates. The distributions of identified good targets also differed significantly between the three schemes (Fig. 2, adjusted *P*-value below 0.05 for all comparisons, Dunn test): cgMLST_V1_ median 97.9% [interquartile range (IQR) 97.6%–98.8%], cgMLST_Biotypes_ median 96.9% (IQR 96.7%–97.7%), and cgMLST_QC_ median 96.8% (IQR 96.6%–97.6%). All non-*Y*. *enterocolitica* species yielded less than 50% good targets across all schemes, indicating that all schemes are specific to *Y. enterocolitica* (Fig. S2). The discriminatory power (Simpson diversity index) across the three schemes was similar: 0.999 [confidence interval (CI) 0.999–1] for cgMLST_V1_ and cgMLST_QC_ and cgMLST_biotypes_ 0.999 (CI 0.998–1).

**Fig 3:**
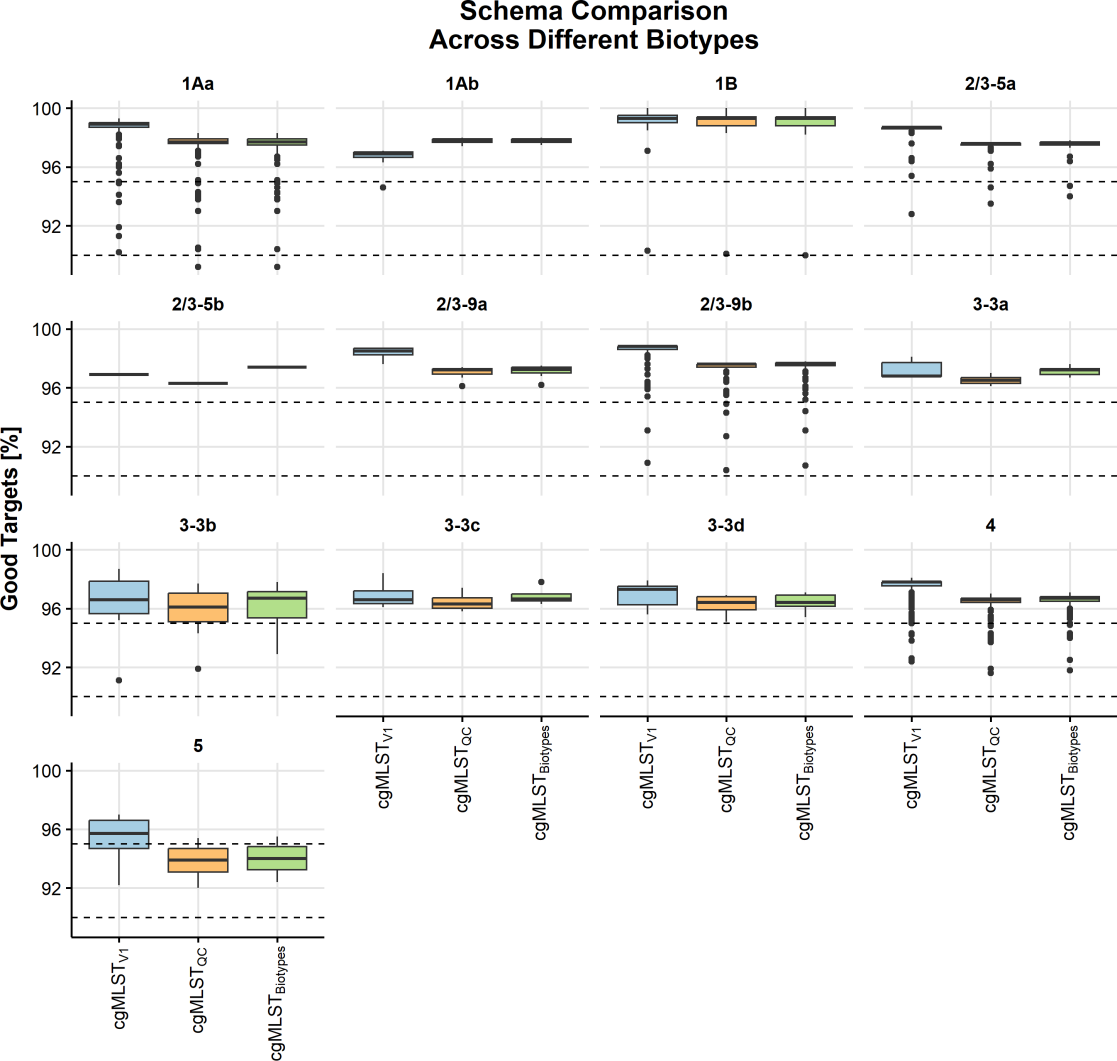
Performance comparison of percentage good targets identified across the three cgMLST schemes (cgMLST_V1_, cgMLST_QC_, and cgMLST_Biotypes_) against the 13 biotype sublineages of *Y. enterocolitica* (1Aa*,* 1Ab, 1B, 2/3–5a, 2/3–5b, 2/3–9a, 2/3–9b, 3–3a, 3–3b, 3–3c, 3–3d, 4, and 5).

We evaluated the performance of the schemes across the different biotypes (Fig. 3). We could not map the biotype of four isolates since they had less than 90% good targets in cgMLST_V1_. Only two biotypes did not achieve at least 95% of the targets in 95% of the isolates across all the schemes: 3–3b (cgMLST_V1_ 85.7% and 71.4% for both cgMLST_QC_ and cgMLST_Biotypes_) and 5 (68.8%, 12.5%, 6.3%, for cgMLST_V1_, cgMLST_Biotypes_, and cgMLST_QC_, respectively). Biotype 1Ab did not achieve this criterion for cgMLST_V1_ (93.8%), whereas 1Aa did not achieve this in both cgMLST_Biotypes_ and cgMLST_QC_ (94.7% each). All biotypes had an IQR above 95%, except for biotype 5, which was below 95% good targets in both cgMLST_QC_ and cgMLST_Biotypes_. Due to this, we identified that cgMLST_V1_ was the best performing scheme and proceeded using this scheme only for the rest of the analysis.

### cgMLST evaluation

We additionally compared the performance of cgMLST on genomes assembled with Velvet, SKESA, and SPAdes (Fig. S3). Both Velvet (median 97.9%, IQR 97.7%–98.7%) and SKESA (median 97.8%, IQR 97.7%–98.8%) performed slightly better compared to SPAdes (median 97.9%, IQR 97.7%–98.7%, Dunn test-adjusted *P*-value 0.0334 and 0.000814, respectively). No difference was observed between SKESA and Velvet assemblies. While there are few differences among these assemblers in terms of the number of target genes identified from these assemblies, SPAdes had assembled more genomes incorrectly leading to some outliers in the percentage of good targets found (Fig. S3). Isolates retrieved from NCBI had a median of 98.4% of the gene targets (IQR 97.3%–98.8%, Fig. S3).

### cgMLST performance within outbreak contexts

**Fig 4:**
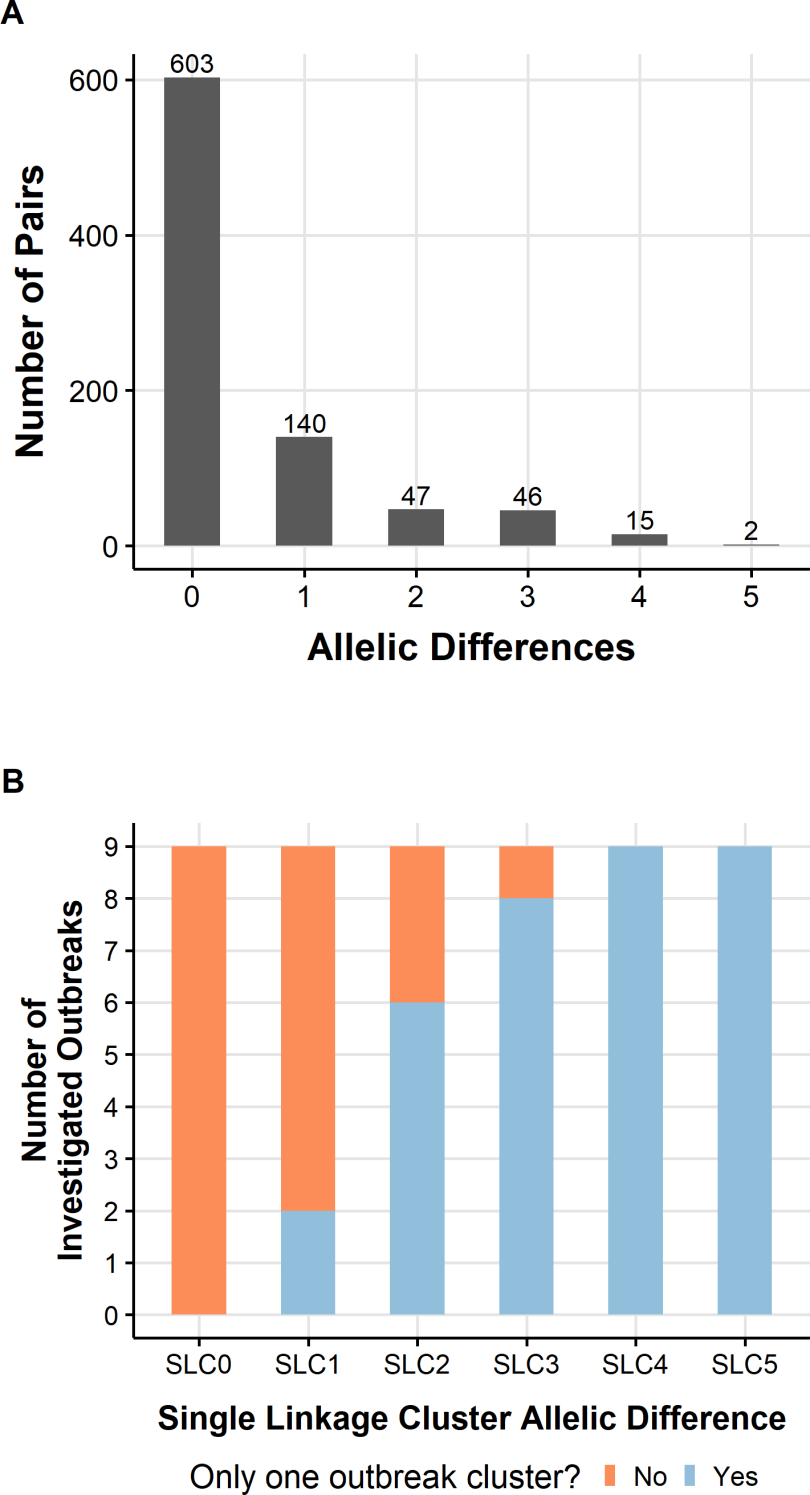
(A). Within outbreak pairwise allelic differences across nine identified outbreaks in Norway between 2018 and 2023. (**B).** Number of investigated outbreaks with only one outbreak cluster using different SLC threshold methods. Each integer after the SLC indicates the maximum allelic differences to cluster isolates.

**Fig 5:**
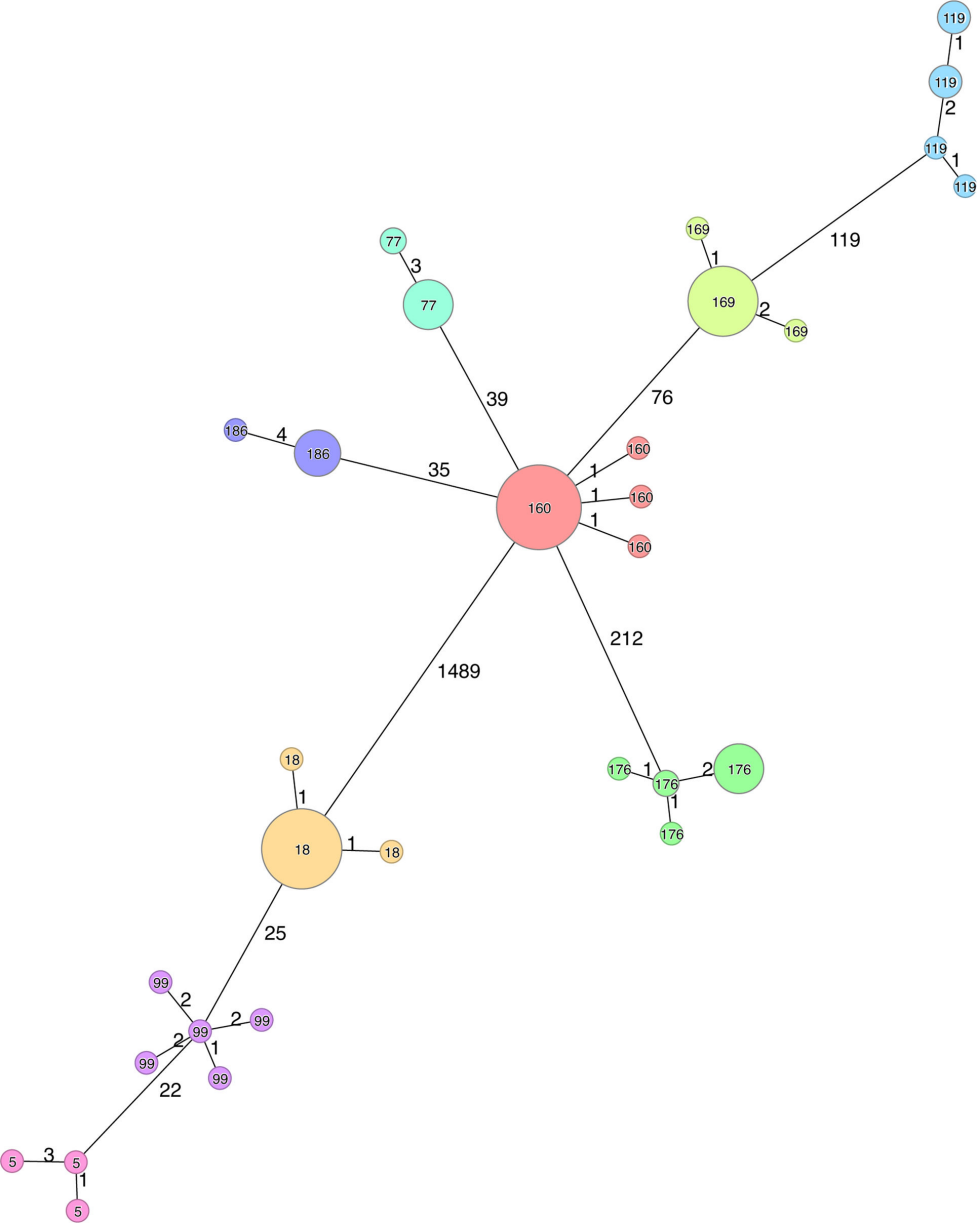
Minimum-spanning tree of all *Y. enterocolitica* outbreak isolates from Norway between 2018 and 2023, using SLC. Numbers inside nodes represent the SLC cluster at a threshold of four allelic differences. Numbers on lines represent the number of allelic differences between isolates.

**Fig 6:**
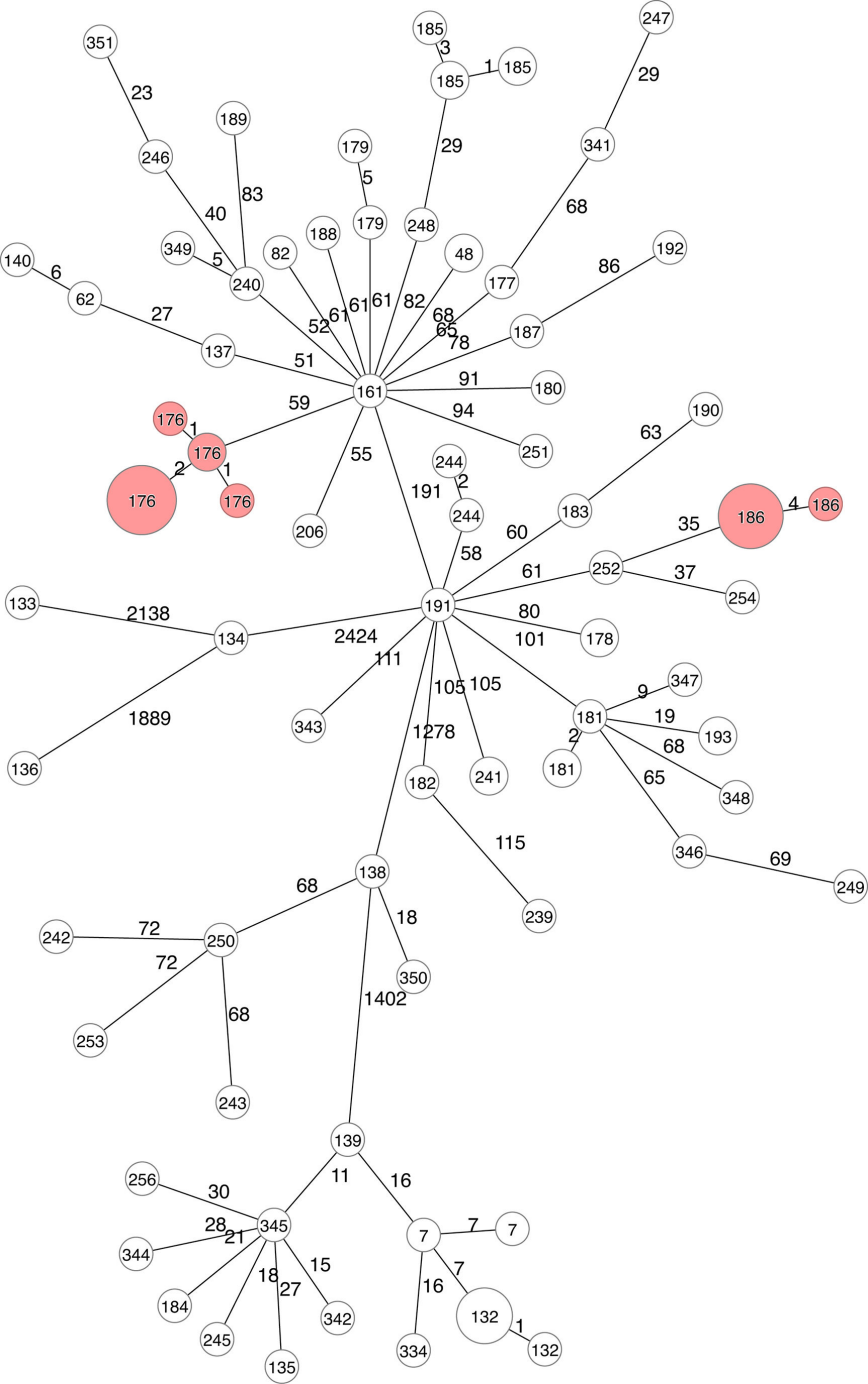
Minimum-spanning Tree of all *Y. enterocolitica* isolates sequenced at NRL in 2022. Nodes in red represent outbreaks. Numbers inside nodes represent the SLC cluster at four allelic differences. Numbers on lines represent the number of allelic differences.

The final cgMLST was also investigated for its usefulness within outbreak scenarios. We investigated retrospectively nine known outbreaks in Norway to identify the number of allelic differences that would group the isolates as the outbreak clone (Table S1; Fig. 4A). Across all the outbreaks, the range of pairwise AD differences ranged between 0 and 5 with a median of 0 (IQR 0–1). However, the SLC threshold, including all isolates within the same SLC, was at 4 AD. Therefore, we established 4 AD as the outbreak threshold. We tested whether using 5 AD would increase the number of isolates within each outbreak cluster, but no additional isolates were added to the different clusters. A minimum-spanning tree of all investigated outbreak isolates (Fig. 5) and isolates from NRL in 2022 (Fig. 6) showed the genetic diversity in cluster types and how the outbreak isolates are clustered into their own CT cluster (Fig. 4B).

When using the outbreak threshold to retrospectively screen the *Y. enterocolitica* received at NRL, as expected, all nine outbreaks used to establish a threshold were identified. In addition, 12 potential outbreak clusters with three or more isolates (threshold for a warning signal) were identified, of which five had five or more isolates (monitoring threshold). After closely inspecting the NIPH monitoring logs, these five clusters were picked as signals by the infection control unit at NIPH, but were not formally investigated as no additional isolates were identified within the next 30 days.

### Comparison with available schemes

We compared the distributions of identified good targets between cgMLST_V1_ and published cgMLST schemes. The Enterobase scheme has 1,553 targets and a median 97.8% (IQR 97.7%–97.9%) good targets were identified (Fig. S3). The Pasteur scheme included 500 gene targets and a median 96% (IQR 95.8%–96%) good targets were identified (Fig. S3). The distribution of good targets differed significantly between all schemes: cgMLST_V1_ and Enterobase (adjusted *P* = 6e−13), cgMLST_V1_ and Pasteur (adjusted *P* = 1.32e−166), and Enterobase and Pasteur (adjusted *P* = 9.51e−168). When comparing the number of unique clusters obtained at different AD thresholds, we observed that Pasteur scheme produces fewer clusters compared to the other schemes (Fig. S4). Both cgMLST_V1_ and Enterobase have similar number of clusters within the first 10 SLCs, but cgMLST_V1_ has a consistently higher of number of clusters compared to Enterobase (Fig. S5A). The number of unique clusters between the three schemes start converging around SLC100 (Fig. S5B).

**Fig 7:**
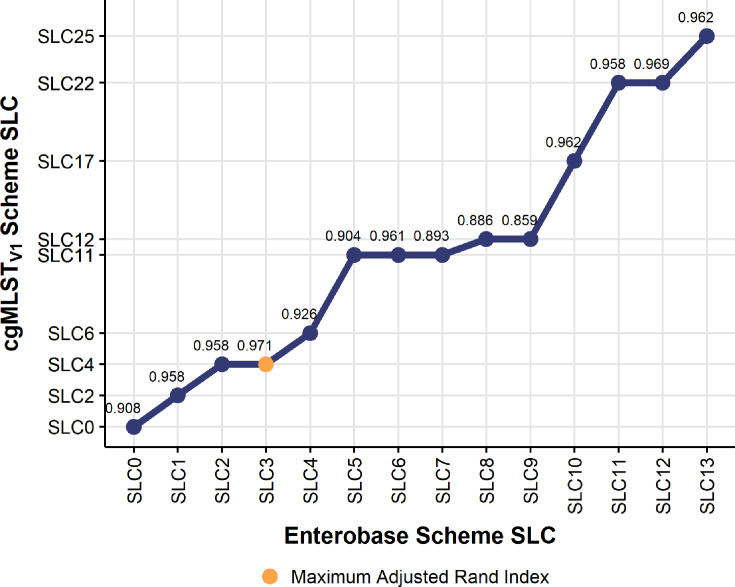
Comparison with SLC cluster assignments between Enterobase SLC at thresholds varying between 0 and 13 allelic differences. At each Enterobase SLC definition, we map to the cgMLST_V1_ SLC obtaining the maximum adjusted Rand index for that comparison. Values for the adjusted Rand index are indicated above the SLC comparison. The maximum value of the adjusted Rand index across all comparisons is marked in orange.

Further, we compared the use of the schemes within outbreak contexts (Fig. S6 and S7). For the Enterobase scheme, the range of pairwise AD differences ranged between 0 and 4 with a median of 0 (IQR 0–0). The SLC threshold, including all isolates within the same SLC, was at 3 AD. For the Pasteur scheme, the range of pairwise AD differences ranged between 0 and 1 with a median of 0 (IQR 0–0). The SLC threshold, including all isolates within the same SLC, was at 1 AD. Given the low resolution of the Pasteur scheme, we only compared SLC matching between Enterobase and cgMLST_V1_. As seen in Fig. 7, the best SLC matching threshold between the two schemes for outbreak detection is SLC4 for cgMLST_V1_ and SLC3 for Enterobase (adjusted Rand index 0.971). However, close inspection of the graph shows a loss of resolution for the Enterobase scheme with increasing numbers of ADs. This is particularly noticeable between 4 and 5 AD of the Enterobase scheme, for which there is a jump in 5 AD in relation to the cgMLST_V1_ scheme.

### SLC clusters in Norway and in public genomes

The NRL isolates were typed into 311 CTs and 22 different sequence types (ST). ST18 was the most frequent sequence type identified (*n* = 329) in Norway. Within this ST, the most common CTs were all part of known outbreaks: CT160 (*n* = 28, outbreak in 2020, associated with spinach), CT169 (*n* = 17, national outbreak in 2021, unknown source), CT176 (*n* = 13, outbreak in 2022, unknown source), and CT77 (*n* = 12, outbreak in 2022, associated with salad). ST12 was the second most frequent ST recovered in Norway (*n* = 109). Within this ST, we also identified that the most frequent CT, CT18 (*n* = 21), - mostly comprised of isolates associated with an outbreak in 2018 where the suspected vehicle was pre-packed salad. Other CTs within ST12 included CT132 (*n* = 9), which has been recovered in recent years (2021–2022), and CT7 (*n* = 8), which has been isolated in multiple years since 2015.

From the public data set, we identified 39 CTs, 22 of which with more than two isolates, including CT261 (*n* = 9) and CT257 (*n* = 6) from Brazilian pigs, CT265 from South Africa (*n* = 6), and CT200 from pigs from Cote d’Ivoire. We also identified the same CT in different countries, such as CT96 recovered from the USA and Belgium, as well as CT184 recovered from both Norway and Germany.

## DISCUSSION

cgMLST schemes have gained increased importance within public health as they provide high resolution and a common nomenclature that facilitate tracking and comparing genomes from different settings (18). However, to achieve this, they need to perform well across the genetic diversity of the species, have high discriminatory power within outbreak investigations, and allow for comparisons spanning different countries and time periods (18, 37, 38). In this study, we developed and evaluated a stable cgMLST scheme for *Y. enterocolitica* and demonstrated its usefulness in the context of outbreak detection and investigation as well as surveillance.

In the development of this scheme, we used the reference strain NC_008800.1 as it was sequenced using Sanger sequencing, which has a lower error rate than NGS (22). Although this is technically more cumbersome, NGS can introduce sequence artifacts due to sequencing errors and assembly. We developed three different schemes and identified the best performing scheme. Ideally, a cgMLST should include the diversity of the lineages of a given species to reduce the identification of targets that appear in a very small number of strains. However, full genomes or chromosomes available through NCBI did not include representatives of each biotype sublineages. The additional two schemes we developed using scaffolds with additional lineages did not improve the performance of the overall scheme, although the number of gene targets decreased. We suspect that genomes with higher contig numbers might preclude the proper identification of some gene targets as genes might be split across multiple contigs. This indicates that the inclusion of good quality genomes might play a more important role than including the entire genetic diversity, as long as enough diversity is included in the development of the scheme.

After evaluating the different schemes, we chose cgMLST_v1_ as the best scheme and investigated the impact of different assemblers on the performance of the schemes. We identified that Velvet or SKESA performed slightly better than SPAdes. While SPAdes also had good performance, some genomes were assigned different cluster types (*n* = 2) compared to the same genome assembled with Velvet or SKESA. This phenomenon could potentially be associated to lower sequence quality and different algorithms used by the different assemblers. Indeed, we identified that across all the genomes that had less than 90% good targets for any of the assemblers, only four were within the assembly quality parameters established by EFSA/WHO (25, 26). Considering that SLC misallocation only occurred in 0.35% of the *Y. enterocolitica* and that often isolates with poor sequence quality are re-sequenced, this is likely not going to affect the results long term in the choice of assembler.

We proceeded with the evaluation of known outbreaks in Norway to establish the number of allelic differences including all outbreak isolates. We identified that a cut-off of four allelic differences would classify all strains as outbreak strains in the Norwegian context. This has also been suggested as the cut-off value for other Enterobacterales species (17). Nonetheless, establishing outbreak cut-offs will depend on the outbreak, and it could be expected that outbreaks lasting long periods of time can lead to microevolutionary processes, which can lead to an adjustment of the outbreak cut-off (39). By using a four allelic difference cut-off, we were able to perform SLC at this level and group isolates. Furthermore, we used this information to retrospectively check for potentially missed outbreaks in the NRL collection. All the nine outbreaks that were investigated by the NIPH and 12 potentially missed outbreaks (signals consisting of clusters of three or more isolates) were identified. Closer inspection of these potentially missed outbreaks indicated that these signals were picked by the NRL, but no formal outbreak investigation was initiated since no further cases of the same CT were reported within a 30-day window. Overall, this shows that our scheme can be used for surveillance and in detection of outbreaks. However, SLC definitions should be adapted to the type of study and outbreak investigation. In Norway, most of the outbreaks have happened within a short period, and thus, there is less expectation of major changes to the genome of the outbreak strain over this time. Nonetheless, outbreaks spanning large periods of time might require relaxing the SLC threshold to accommodate case and/or source finding, and the increased resolution of our scheme allows to set these thresholds at diverse AD.

When comparing our scheme to the published schemes available—Enterobase and Pasteur (8, 17), our analysis indicates that the developed scheme performs better compared to the other schemes. Both our scheme and the Enterobase scheme perform better than the Pasteur scheme in terms of identified gene targets and resolution. Between Enterobase and cgMLST_V1_, the difference in the median is minute, and the statistical difference is likely due to the higher 75th quantile of the distribution being higher for cgMLST_V1_ compared to Enterobase. However, the advantage of cgMLST_V1_ over Enterobase is also observed in the level of resolution in terms of outbreak investigations. As seen in Fig. 4 and 3, there is 1 AD difference in terms of the applied outbreak investigation threshold. While this seems like a small difference, SLC definitions need to be established for each outbreak investigation. Taking this into consideration, we can see that the Enterobase scheme rapidly loses granularity compared to cgMLST_V1_ when increasing the AD threshold for SLC. This is particularly important in cross-border outbreaks where the number of AD can be much higher than for local outbreaks. For some Enterobacterales species, this can be set at 10 AD (34–36). Based on this working threshold, we observed that Enterobase after SLC at 5 AD have a correspondence with cgMLST_V1_ at 11 AD and above. This shows the higher resolution of cgMLST_V1_ compared to Enterobase. This increased resolution of cgMLST_V1_ allows for increased flexibility in establishing SLC for different purposes—outbreak or surveillance—and enables public health institutions to better assess the need to intervene and deploy resources in an event of a potential outbreak.

Finally, we applied the SLC to all isolates at the NRL and public databases. While many of the CTs common in Norway have been associated with outbreaks, we could also identify that our scheme can track CT across different years. The identification of CTs over time is important for tracking other processes, including CTs with increased pathogenicity or sporadic infections linked to the same source spread across time. Overall, this confirms that our developed scheme is useful in both outbreak investigations as well as for surveillance. Comparing our data with genomes in NCBI, we identified that the population structure is diverse. Furthermore, we identified a similar CT within the same country but also between countries (Table S1). This indicates that *Y. enterocolitica* has some degree of geographic specificity, but also the potential to be useful in cross-border outbreaks, as previously reported (7). Altogether, this suggests that this scheme could be implemented in other countries.

Data from the literature also indicate that ST18 and ST12 are commonly recovered from humans and pigs in distinct geographical regions, including the United Kingdom (40), Latvia (41), New Zealand (16), and Brazil (42). Although it was not the goal of this study to provide a picture of the global molecular epidemiology of *Y. enterocolitica*, our scheme has identified several CTs within frequent STs. Hence, future studies applying our scheme in different settings will provide a better idea of the population structure of *Y. enterocolitica* and uncover potential clonal geographic overlaps.

### Limitations

The low representation of some biotype sublineages in the development and evaluation yields some uncertainty on the real performance of the scheme on these linages. We attempted to resolve this issue by supplementing our data set with genomes available in NCBI, but the representation of these lineages herein was also very low. Nonetheless, we demonstrated that the percentage of good targets were above 90% in all biotype sublineages, and thus, this scheme is suitable for comparison across genomes. Second, the evaluation of the scheme’s performance within outbreaks was only possible for Norwegian isolates. However, considering that we could identify similar SLC types within different geographic regions, we expect that this scheme will perform well in different countries. Third, we could not test this scheme on isolates from all outbreak sources. Nonetheless, we observed for the 2014 outbreak that the salad isolates are considered part of the same outbreak already at SLC1 and for the 2022 outbreak (pork product), at SLC2 (data not shown due to data ownership). Fourth, all the NRL genomes used were sequenced using short sequencing technology, which can be difficult to assemble and may generate errors especially for repetitive regions, which are present in *Y. enterocolitica* (22, 43). Nevertheless, differences in CT allocation were observed for a very small number of isolates (*n* = 2) just for one assembler suggesting that using assemblies from short reads for the developed cgMLST scheme is likely not an issue. Finally, the number of allelic differences does not equate to the number of single nucleotide polymorphisms between isolates leading to loss in resolution. Despite this fact, several studies have demonstrated the congruence of SNP and cgMLST schemes for other species and recommended the usage of cgMLST given the possibility to generate a stable comparable nomenclature (44, 45).

### Conclusion

The developed cgMLST scheme for *Y. enterocolitica* has shown to have high discriminatory power and perform well across the genetic diversity of *Y. enterocolitica*. The scheme has also identified outbreak strains, and the same complex types may be linked to national and international spread. We recommend the implementation of this scheme in public health institutions to improve the surveillance and outbreak management of this pathogen.

## Data Availability

All genomes used in this study have been deposited to the European Nucleotide Archive (ENA), and 431 are available through BioProject PRJEB67986. The cgMLST scheme is available at https://cgmlst.org/ncs.

## SUPPLEMENTAL MATERIAL

Supplemental material jcm.00040-24-s0001.pdf Figures S1 to S7; legends for Tables S1 and S2.

Table S1 jcm.00040-24-s0002.csv Metadata of genomes used in the study.

Table S2 jcm.00040-24-s0003.csv Characteristics of seed genomes used in the development of cgMLST scheme.

ASM does not own the copyrights to Supplemental Material that may be linked to, or accessed through, an article. The authors have granted ASM a non-exclusive, world-wide license to publish the Supplemental Material files. Please contact the corresponding author directly for reuse.
