# Supplementary material for: Development and implementation of a core genome multilocus sequence typing scheme for Yersinia enterocolitica: a tool for surveillance and outbreak detection
Source: J Clin Microbiol. 2024 Jul 11;62(8):e00040-24. doi: 10.1128/jcm.00040-24 (PMC11325262; doi:10.1128/jcm.00040-24)
Supplement: Supplemental material — Figures S1 to S7; legends for Tables S1 and S2. [file jcm.00040-24-s0001.pdf]

Supplementary Information for:

Development and Implementation of a Core Genome Multilocus Sequence Typing scheme for *Yersinia enterocolitica:* A Tool for Surveillance and Outbreak Detection

Joao Pires[1,2], Lin T. Brandal[1], Umaer Naseer[1*]

[1] Department of Infection Control and Preparedness, Norwegian Institute of Public Health, Norway

[2] ECDC Fellowship Programme, Public Health Microbiology path (EUPHEM), European Centre for Disease Prevention and Control (ECDC), Stockholm, Sweden

*Corresponding author. E-mail: mohammed.umaer.naseer@fhi.no

Supplementary Figures

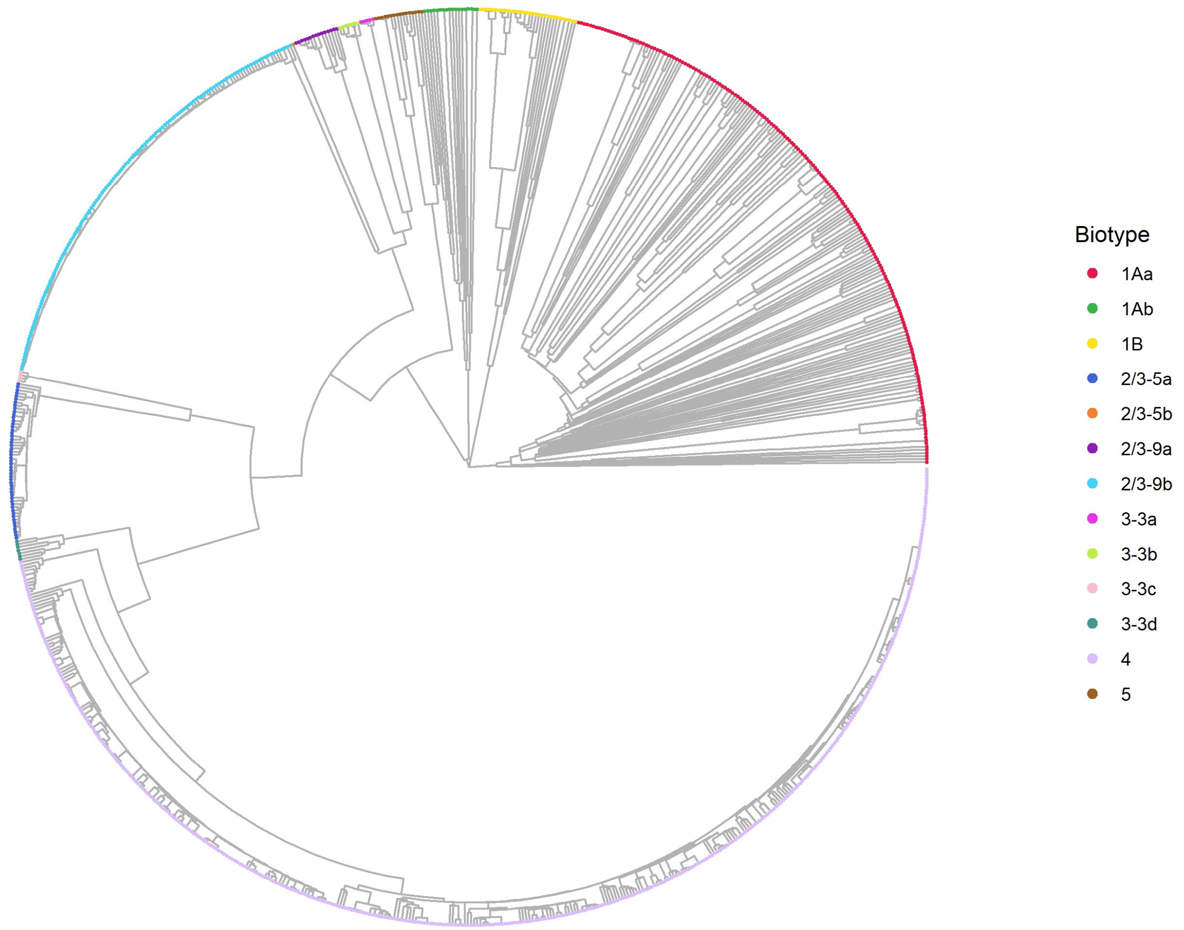

Figure S1. UPGMA tree of all isolates included in the study. Biotypes have been putatively assigned based on isolates from Savin et al. (1).

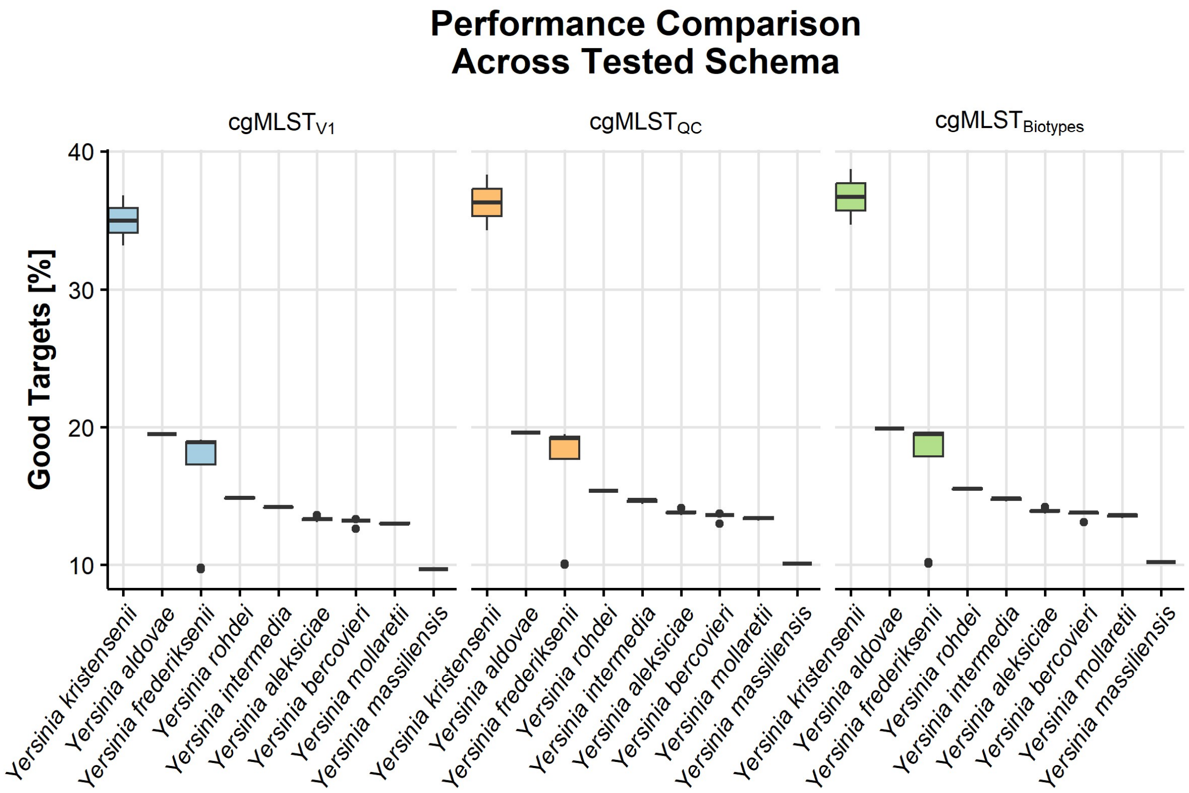

Figure S2. Evaluation of the cgMLST schema on non-*Y. enterocolitica* species.

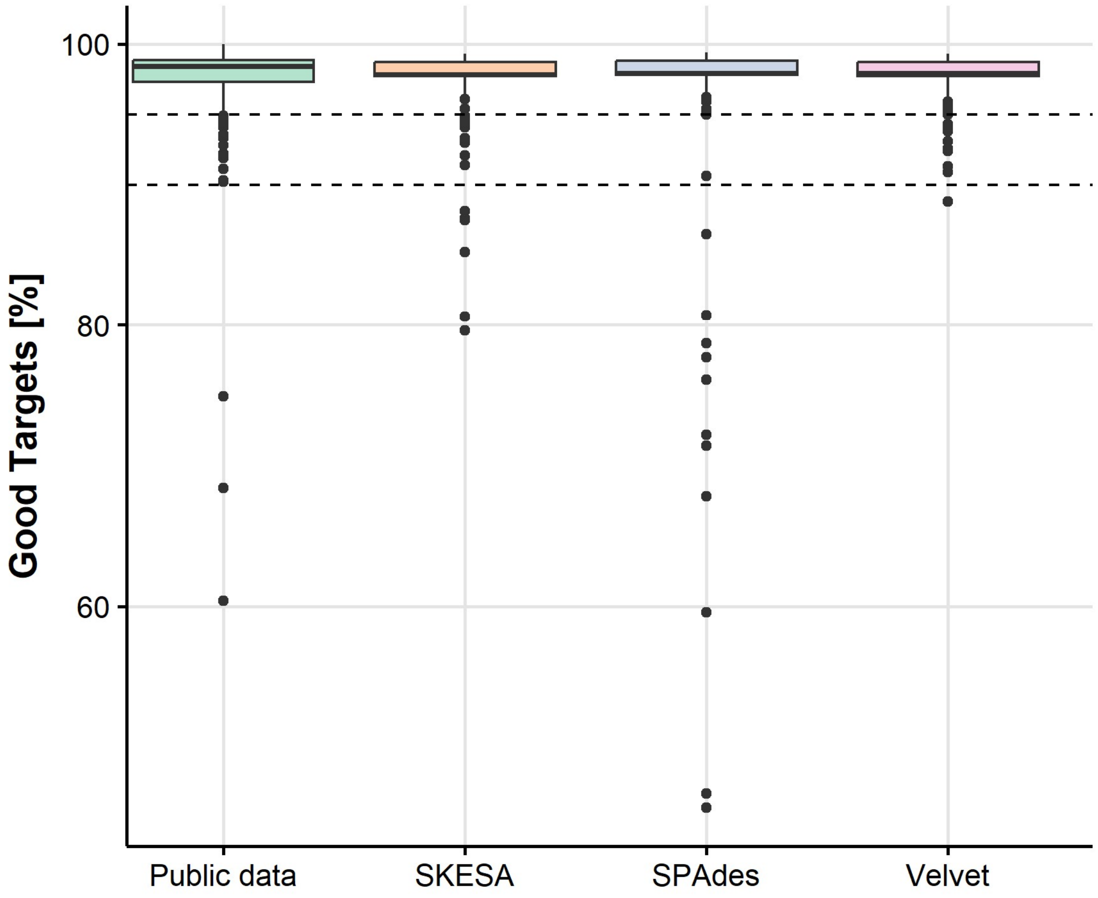

Figure S3. Performance comparison across assemblers.

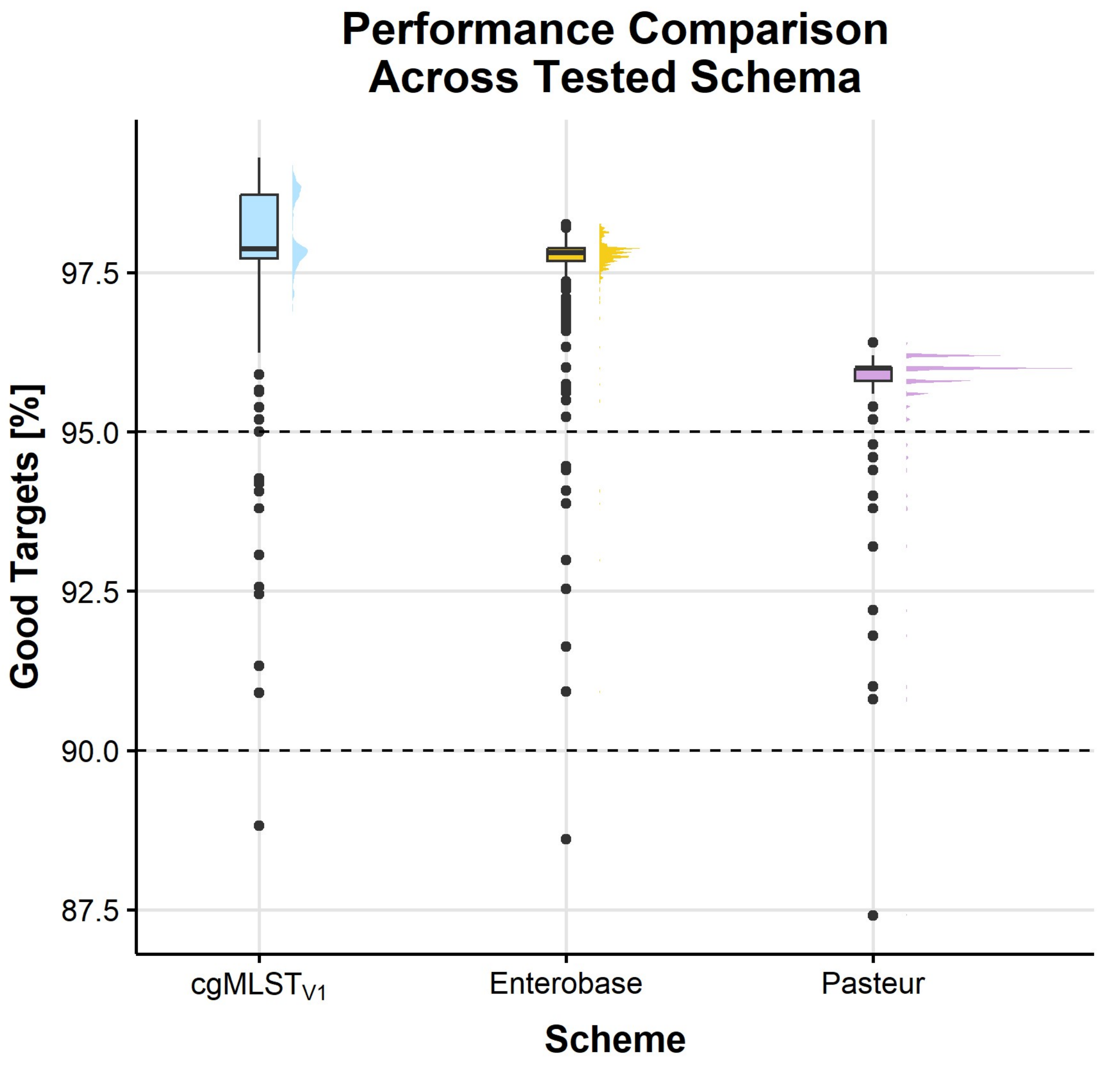

Figure S4. Performance comparison of percentage good targets identified across cgMLST schemes ($cgMLST_{V1}$, Enterobase and Pasteur). The $cgMLST_{V1}$ scheme has 2582 targets and a median of 97.9% (interquartile range [IQR] 97.6%-98.7%) good targets; the Enterobase scheme has 1553 targets and a median 97.8% (IQR 97.7-97.9%) good targets; the Pasteur scheme included 500 gene targets and a median 96% (IQR 95.8%-96%) good targets.

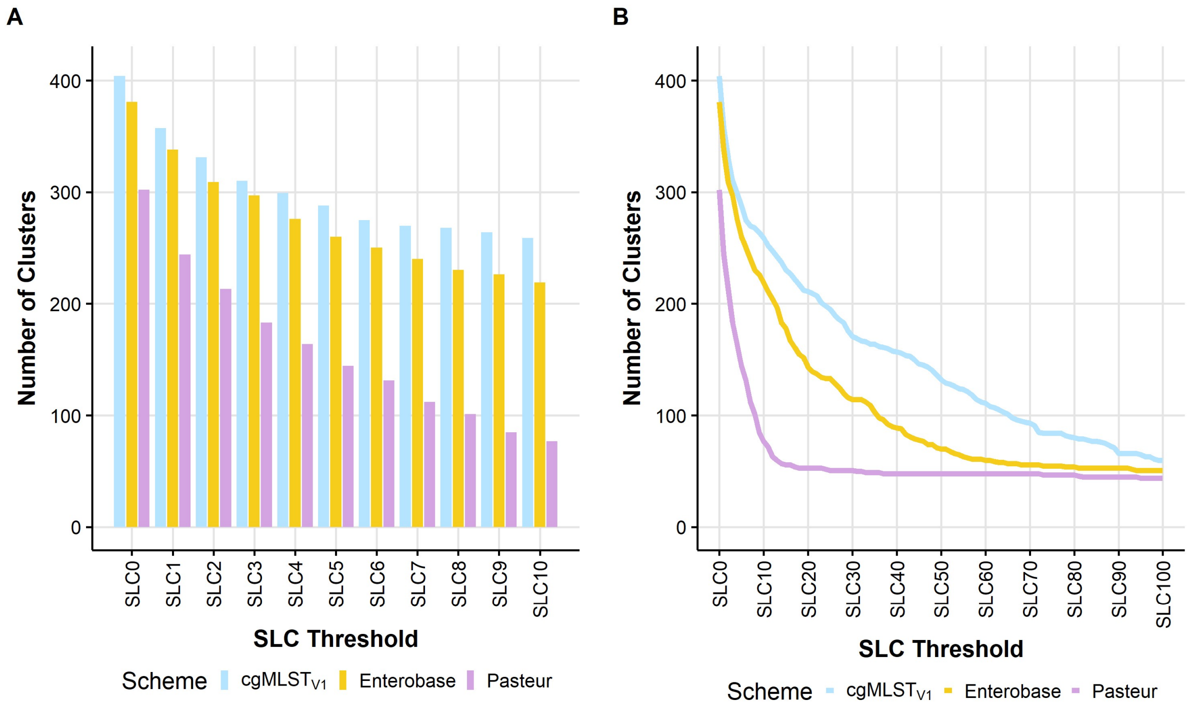

Figure S5. Number of distinct cluster types obtained at different SLC clustering thresholds for the different cgMLST schemes. **A.** Shows number of cluster zoomed in the "outbreak investigation" working zone (SLC 0 – SLC 10). **B.** Shows number of clusters up to SLC 100.

Enterobase Scheme

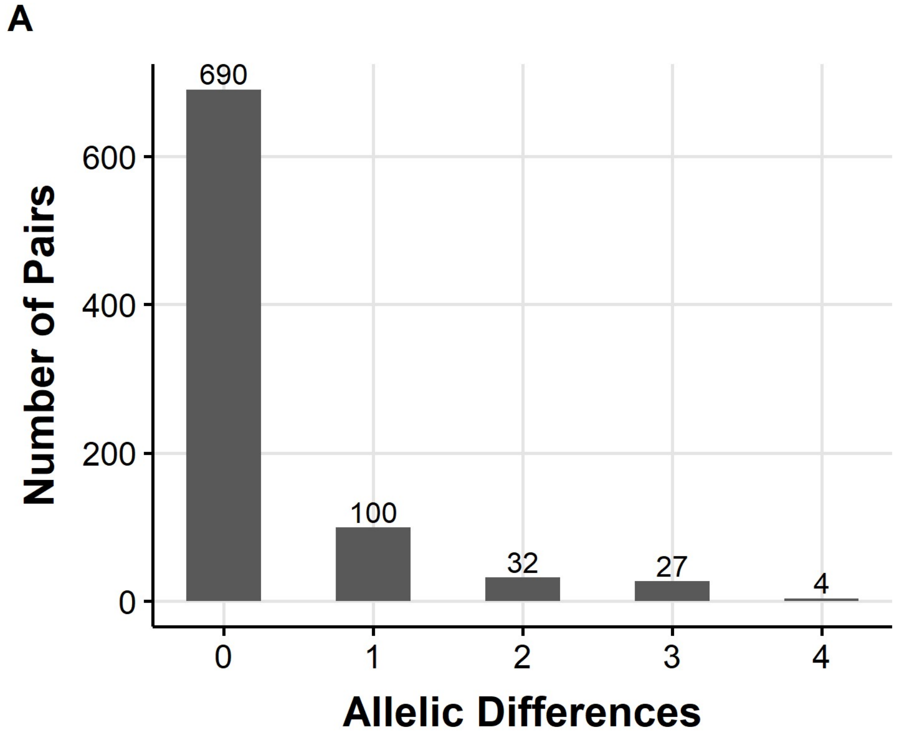

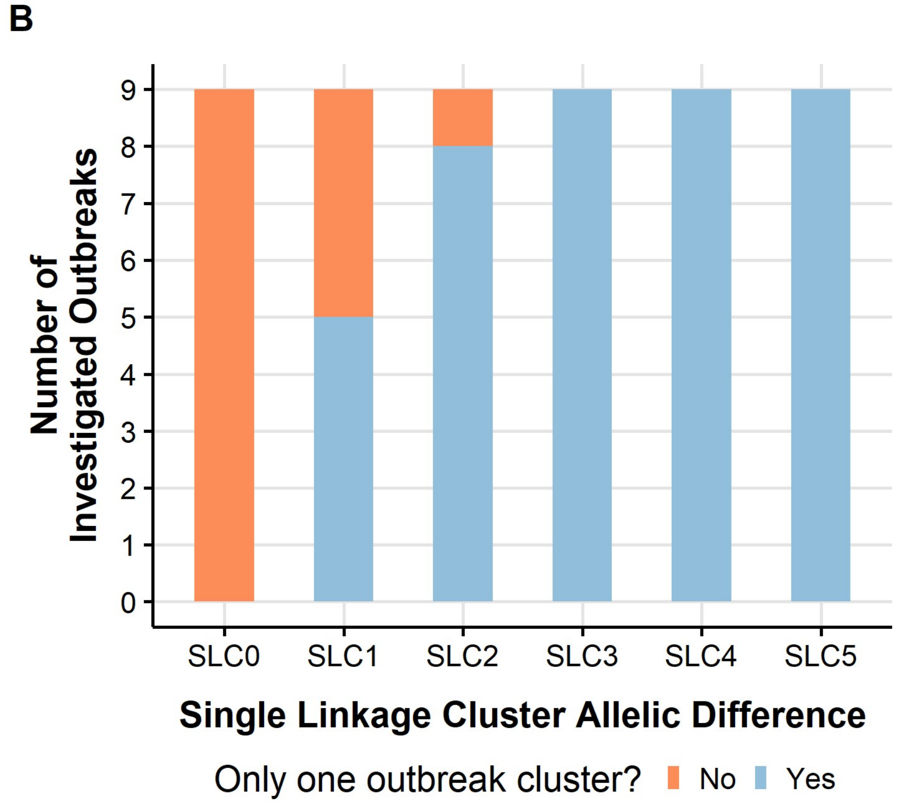

Figure S6. A. Within outbreak pairwise allelic differences across 9 identified outbreaks in Norway between 2018-2023. **B.** Number of investigated outbreaks with only one outbreak cluster using different Single-Linkage Clustering (SLC) threshold methods. Each integer after the SLC indicates the maximum allelic differences to cluster isolates.

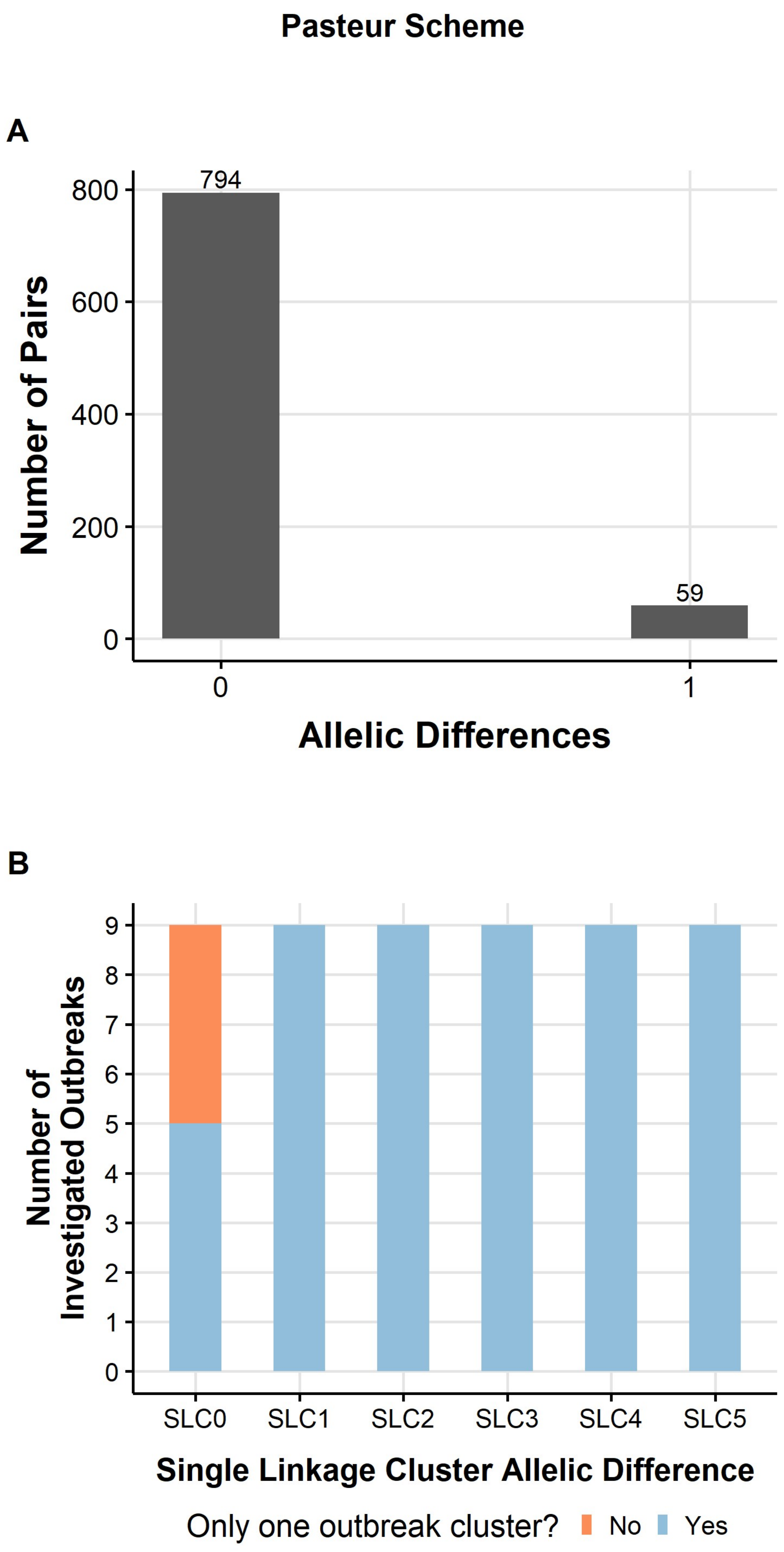

Pasteur Scheme
A
794
59
800
600
400
200
0
Number of Pairs
0
1
Allelic Differences
B
9
8
7
6
5
4
3
2
1
0
Number of Investigated Outbreaks
SLC0
SLC1
SLC2
SLC3
SLC4
SLC5
Single Linkage Cluster Allelic Difference
Only one outbreak cluster?
No
Yes

Figure S7. A. Within outbreak pairwise allelic differences across 9 identified outbreaks in Norway between 2018-2023. **B.** Number of investigated outbreaks with only one outbreak cluster using different Single-Linkage Clustering (SLC) threshold methods. Each integer after the SLC indicates the maximum allelic differences to cluster isolates.

Supplementary Table Legends

Table S1 – Metadata of isolates included in this study

Sample_ID – isolate identification number. Isolates starting with "NIPH" indicate isolates sent to the Norwegian Institute of Public Health National Reference Laboratory. For isolates from public repositories, the Sample_ID corresponds to their BioSample identifier

Species – bacterial species identified

Biotype – putative biotype as defined by Savin et al. (1).

Date – isolation date if available

Country – isolation country if available

Source – isolation host if available

ST – *Y. enterocolitica* sequence type if available

Serotype – *Y. enterocolitica* if available

SRA – Sequence Read Archive identifier. Only available for "NIPH" isolates

Year – Isolation Year

cluster_id – code assigned by the Norwegian Institute of Public Health for outbreak designation

Vehicle – putative vehicle for outbreak isolates

SLC_1 – single linkage cluster at 1 allelic difference assigined by SeqSphere+

SLC_2 – single linkage cluster at 2 allelic differences assigined by SeqSphere+

SLC_3 – single linkage cluster at 3 allelic differences assigined by SeqSphere+

SLC_4 – single linkage cluster at 4 allelic differences assigined by SeqSphere+

SLC_5 – single linkage cluster at 5 allelic differences assigined by SeqSphere+

Table S2 – Seed Genome Characteristics

BioSample – BioSample identifier

Biotype – putative biotype as defined by Savin et al. (1).

Assembly_Genbank_Id – Assembly GenBank identifier

Assembly_ RefSeq_Id - Assembly RefSeq identifier

Status – genome assembly status

Coverage – genome coverage

N50 – N50 value

N_Contigs – number of contigs

cgMLSTv1 – whether seed genome was used in the design of $cgMLST_{v1}$ scheme

cgMLSTQC – whether seed genome was used in the design of the cgMLSTqc scheme

cgMLSTbiotypes – whether the seed genome was used in the design of cgMLSTBiotypes scheme
